# Indoor distribution characteristics of airborne bacteria in pig buildings as influenced by season and housing type

**DOI:** 10.5713/ajas.18.0415

**Published:** 2018-08-27

**Authors:** Ki Youn Kim, Han Jong Ko

**Affiliations:** 1 Department of Safety Engineering, Seoul National University of Science & Technology, Seoul 01811, Korea; 2 Department of Agricultural Sciences, Korea National Open University, Seoul 03087, Korea

**Keywords:** Airborne Bacterial, Pig Building, Gestation/Farrowing, Nursery, Growing, Fattening

## Abstract

**Objective:**

A concentration of airborne bacteria generated from swine houses is recognized to be relatively higher than other work places and it is essential to optimally manage it to prevent farmers’ respiratory diseases. This study was conducted to assess the distribution characteristics of airborne bacteria in swine houses located at South Korea.

**Methods:**

A total 27 pig buildings of the enclosed type operated with mechanical ventilation system by a side wall fan and deep-pit manure system with slats were surveyed. Air samples were collected at 1.0 m above the middle floor in pig housing room. A six-stage viable particulate cascade impactor was used to identify the distribution of the sizes of particles in diameter.

**Results:**

Seasonal mean levels of airborne bacteria in the housing rooms of gestation/farrowing pigs, nursery pigs and growing/fattening pigs were 3,428(±1,244) colony forming unit (cfu)/m^3^, 8,325(±3,209) cfu/m, and 13,254(±6,108) cfu/m^3^ for spring; 9,824(±2,157) cfu/m^3^, 18,254(±5,166) cfu/m^3^, and 24,088(±9,274) cfu/m^3^ for summer; 1,707(±957) cfu/m^3^, 4,258 (±1,438) cfu/m^3^, and 8,254(±2,416) cfu/m^3^ for autumn; and 2,322(±1,352) cfu/m^3^, 6,124(±1,527) cfu/m^3^ and 12,470(±4,869) cfu/m^3^ for winter, respectively.

**Conclusion:**

Concentrations of airborne bacteria according to pig housing type were highest in growing/fattening housing room followed by nursery housing room and gestation/farrowing housing room. In terms of seasonal aspect, the pig building showed the highest levels of airborne bacteria in summer followed by spring, winter and autumn. The respirable airborne bacteria which are ranged between 0.6 and 4.7 μm accounted for approximately 60% compared to total airborne bacteria regardless of pig housing type.

## INTRODUCTION

Concern and regulations on environmental pollution and health problems are growing as the livestock production systems are transformed to a limited and commercialized type. Although difficult, it is urgent to deal with the situation because the air pollutants derived from livestock farming practice can affect a very wide range of areas [1,2].

Currently, most pig buildings situated in South Korea are operated by an automatic mechanical ventilation system to promote pig productivity through optimal thermal control and requiring a smaller work force. Most notably, however, these pig buildings are not equipped with adequate ventilation technology suitable for the local climatic conditions as well as effectively applying efficient ventilation systems to minimize the management cost. However, it is almost impossible to sustain an appropriate working environment in open pig buildings without mechanical ventilation are because they are also operated under poor economic conditions. Therefore, the indoor environment in the pig building contains considerable amounts of air pollutants due to inadequate ventilation and can be a serious health hazard to both farmers and pigs [3,4]. Especially airborne microbes combined with solid particles can expose farmers and pigs to infectious and allergic diseases such as pneumonia, asthma and rhinitis [5–8].

Foreign studies related to indoor airborne bacteria were conducted for not only pig buildings but also residential homes, multiuse facilities and general work sites. These reports show that concentration of airborne bacteria in pig buildings is relatively higher than other indoor areas [4,9,10]. The studies regarding airborne bacteria in pig buildings were mainly conducted in terms of hygiene for preventing respiratory disease of farmers and focused on identifying the exposure level of airborne bacteria and the correlation relationship with other air pollutants such as ammonia and dust [3,4,11]. In the E.U., concentration and emission units of airborne bacteria in each country were estimated by quantifying them through a large field survey according to type of pig building [12].

Several studies identified the species of airborne bacteria in the pig building. *Staphylococcus* spp. was a predominant bacteria while *Salmonella* spp. was not detected in the indoor air of pig buildings [13]. Various species of airborne bacteria are distributed in the pig building and most of them were gram positive bacteria whereas gram negative bacteria were present at very low concentrations [14].

Therefore, indoor air pollution in pig buildings by airborne bacteria may be a major cause of decreased pig productivity and respiratory diseases of farmers such as asthma, rhinitis, and bronchitis. Consequently, it is necessary to conduct a fundamental study in South Korea because there is little domestic information regarding the distribution characteristics of airborne bacteria in pig buildings. The objective of this study is to provide basic research data available to environmental management by analyzing airborne bacteria in pig building through on-site investigation reflecting the characteristics of pig buildings and seasonal conditions in South Korea.

## MATERIALS AND METHODS

### Subject

The pig buildings surveyed in this study were an enclosed type operated with a mechanical ventilation system by side wall fan and deep-pit manure system with slats. To wholly represent areas of South Korea which consists of 9 provinces (Gyeonggi, Gangwon, Chungbuk, Chungnam, Gyeongbuk, Gyeongnam, Jeonbuk, Jeonnam, and Jeju), three pig buildings per each province were selected randomly and the total of 27 pig buildings were surveyed in this study as presented in Table 1. In addition, they were visited every month in 2016 and the mean data obtained for three consecutive months were considered as a seasonal level: spring (Mar to May), summer (Jun to Aug), autumn (Sep to Nov) and winter (Dec to Feb) to reflect characteristics of four seasons of South Korea. The sampling sites in pig building were gestation/farrowing room, nursery room and growing/fattening room.

### Measurement

**Figure 1:**
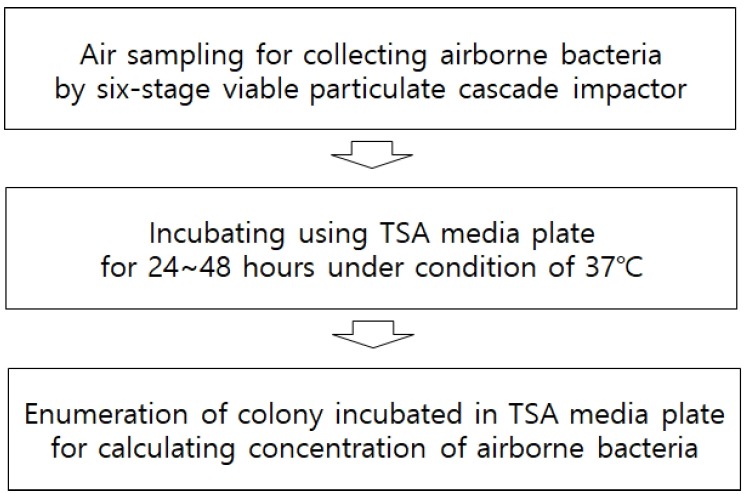
Process of measurement and analysis of airborne bacteria.

Air Samples were collected at 1.0 m above the middle floor in pig housing room. As represented in Figure 1, a six-stage viable particulate cascade impactor (TE-10-800, Tisch Environmental Inc., Cleves, OH, USA) was used to identify the diameter distribution of the sizes of particles. The range of aerodynamic diameters at each stage was: stage 1 (>7.0 μm), stage 2 (4.7 to 7.0 μm), stage 3 (3.3 to 4.7 μm), stage 4 (2.1 to 3.3 μm), stage 5 (1.1 to 2.1 μm), stage 6 (0.65 to 1.1 μm). Collection of samples was carried out at each collection point at 1.5 m above the floor for 10 minutes by sucking the air at the rate of 28.3 L/min. A sterilized media, consisting of trypticase soy agar (TSA) (Lot 3087230, Becton Dickinson and Company, Trenton, NJ, USA), to which was added 500 mg/L of cycloheximide to inhibit the proliferation of fungi, was placed in each stage of the equipment, six-stage viable particulate cascade impactor, for the collection of respective size of particles. Every time a media was replaced for the collection of a different type of microorganism and before starting each collecting operation, the exterior of the collection equipment was sterilized with 70% ethyl alcohol in order to prevent contamination. After collecting an air sample the media was taken out of the equipment and immediately sealed with a film for laboratory use to prevent contamination, and it was directly transported to microbial analysis laboratory where it was cultured for 24 to 48 hours at 37°C before observation.

The concentration of total airborne bacteria was determined by adding the number of colonies cultured from all the stages and was represented with corrected value. The lower limit of detection was 30 colonies cultured in agar media. Each concentration (colony forming unit [CFU]/m^3^) of airborne bacteria for respective particle size was determined by dividing the number of colonies cultured separately for each stage by the sampled air volume (m^3^) (Equation 1, 2).

(Equation 1) $$\text{CFU}/{\text{m}}^{3}=\text{Colony counted on agar plate}/\text{air volume }({\text{m}}^{3})$$

(Equation 2) $$\text{Air volume }({\text{m}}^{3})=28.3\;\text{L}/\text{min}\times \text{sampling time }(\text{min})/10^{3}$$

All the cultured airborne bacteria were identified to the level of genera according to Bergey’s manual, and after Gram staining, a biochemical test was conducted to identify genera with an automatic identification system - VITEK (Model VITEK 32 system, bioMerieux Inc., Marcy I’Etoile, France).

Environmental factors were also measured to analyze correlation with the concentration of bioaerosol existing in the air inside the feedstuff-manufacturing factories. Temperature and carbon dioxide were measured with direct indoor air quality analyzer (SensorLynk, IST Inc, Releigh, NC, USA) and relative humidity was measured using an Assmann ventilated psychrometer (SATO R-704, SATO Inc, Tokyo, Japan).

### Statistics

SAS package (SAS/Stat 9.1, SAS Institute Inc., Cary, NC, USA) program was used for the statistical analysis of measurement data. Using Shapiro-Wilk test, each factor was translated into geometric normal distribution and geometric mean and geometric standard deviation were calculated as representing values. The statistical analysis was applied to verify the significance of concentration difference in airborne bacteria among pig housing rooms (gestation/farrowing room, nursery room and growing/fattening room) and seasons (spring, summer, autumn, and winter).

## RESULTS AND DISCUSSION

### Quantification analysis

As shown in Figure 1, mean concentrations of airborne bacteria in the housing room of gestation/farrowing pigs were 3,428 (±1,244) cfu/m^3^ for spring, 9,824(±2,157) cfu/m^3^ for summer, 1,707(±957) cfu/m^3^ for autumn, and 2,322(±1,352) cfu/m^3^ for winter, respectively. Mean concentrations of airborne bacteria in the housing room of nursery pigs were 8,325(±3,209) cfu/m^3^ for spring, 18,254(±5,166) cfu/m^3^ for summer, 4,258(±1,438) cfu/m^3^ for autumn, and 6,124(±1,527) cfu/m^3^ for winter, respectively. Mean concentrations of airborne bacteria in the housing room of growing/fattening pigs were 13,254(±6,108) cfu/m^3^ for spring, 24,088(±9,274) cfu/m^3^ for summer, 8,254 (±2,416) cfu/m^3^ for autumn, and 12,470(±4,869) cfu/m^3^ for winter, respectively. In terms of pig housing rooms, the growing/fattening room showed the highest level of airborne bacteria followed by nursery room and gestation/farrowing room (p< 0.05). The mean level of airborne bacteria in summer was highest regardless of type of pig housing room (p<0.05). There was, however, no significant difference of airborne bacteria among spring, autumn and winter (p>0.05).

The distribution characteristics of airborne bacteria according to type of pig housing room, which appears through these measurements, are considered to result from the growth phase and activity of the pig. In other words, the body of pigs in the growing/fattening room are huge and their activity is also high because this room is used for rearing pigs before shipment. Thus, the particles of feedstuff and dried manure deposited on floor surface of the pig housing room, which would function as a source of nutrition supply for airborne bacteria, have a relatively high potential to be dispersed in the air. On the other hand, the finding that mean level of airborne bacteria in the gestation/farrowing room was lower than other pig housing rooms would be explained by relatively low pig activity attributed to the fact that pregnant pigs in the gestation/farrowing room are kept in farrowing crates. In seasonal aspect, generally the concentrations of airborne bacteria were the highest in summer and the lowest in winter, which would be due to optimal conditions for bacteria to multiply in summer due to the increase in indoor temperature.

In this field study, only the pig building which consists of an enclosed type operated with mechanical ventilation system by side wall fan and deep-pit manure system with slats was investigated because it is dominant and representative type among pig buildings situated in South Korea. However, differences of pig building type such as ventilation system and manure management would affect the level of airborne bacteria. Thus, further study regarding comparison of airborne bacteria concentration according to the modes of ventilation system and manure management should be conducted in the future.

According to previous reports regarding measurement of airborne bacteria in pig buildings, levels ranged between 104 cfu/m^3^ and 106 cfu/m^3^ [4,11,12,14–20]. However, it is not objective to directly compare these previous results with the results obtained from this study since the investigation period, sampling technique and weather condition of the measurement day were different between the previous studies and this study. In addition, the concentrations of airborne bacteria in pig building reported by this study were estimated to be 10 to 100 times higher as compared to those measured in the general residential facilities [21–24]. The result would be explained by organic materials such as feed, excreta and pig skin, which can be the main source of microorganisms, are relatively large in pig buildings compared to general residential facilities [14].

### Size distribution

**Figure 2:**
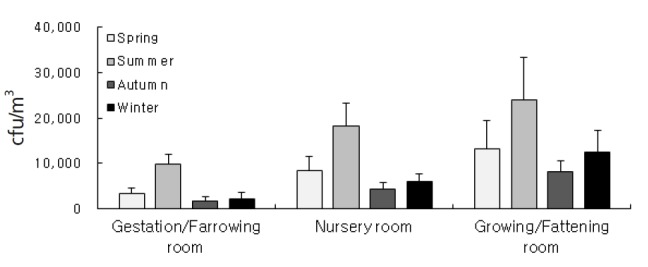
Mean seasonal concentration of airborne bacteria in pig room according to type of pig housing room.

Figure 2 illustrates aerodynamic size distribution of airborne bacteria in the pig building and the results are as follows: stage 1 (19%), stage 2 (23%), stage 3 (22%), stage 4 (20%), stage 5 (10%), and stage 6 (6%) for gestation/farrowing room; stage 1 (18%), stage 2 (22%), stage 3 (20%), stage 4 (19%), stage 5 (10%), and stage 6 (11%) for nursery room; and stage 1 (20%), stage 2 (24%), stage 3 (19%), stage 4 (16%), stage 5 (13%), and stage 6 (8%) for growing/fattening room, respectively. Although the aerodynamic size distribution of airborne bacteria in the pig building was observed to be somewhat different among pig housing rooms, overall airborne bacteria sizes are between 0.6 and 4.7 μm (stage 3 to stage 6), which corresponds to respirable fraction, and was estimated to account for about 60% of the total size.

### Identification

Table 2 shows identification results of airborne bacteria in pig housing rooms of pig buildings. Of all the species of airborne bacteria detected in the study, *Micrococcus* spp., *Brevibacillus* spp. and *G(+) Bacillus* were identified as common in all the pig housing rooms. In general *Micrococcus* spp. showed the highest detection rate followed by *Brevibacillus* spp. and *G(+) Bacillus*, which implies that they are the predominant species among airborne bacteria in the air of pig buildings. The finding that some species were detected in only one pig housing room was unique in this study. These restricted species were *Staphylococcus saprophytius*, for gestation/farrowing room, *Proteus mirabilis* for nursery room and *Aerococcus viridans* for growing/fattening room, respectively.

In the case of previous foreign researches related to identificcation of airborne bacteria in pig building, *Staphylococcus* spp. was a predominant airborne bacteria and *Salmonella* spp., a pathogenic microorganism, was not detected at all [13]. Various microorganisms are distributed in the air of pig buildings and most of airborne bacteria were gram positive bacteria with gram negative bacteria being present at very low concentrations [14]. Additionally they reported that the main predominant species of airborne bacteria in pig building were *Enterobacter agglomerans*, *Moraxella*, *Acinetobacter calcoaceticus*, and *Pseudomonas* spp..

The restriction in this measurement result indicates that the ratio of unidentified species to total airborne bacteria was in the high range of 40% to 60% due to the limitations of the detection technique applied to this study. Therefore, to thoroughly detect species of airborne bacteria in pig buildings, the MIDI technique through analyzing fatty acid of microorganisms or the polymerase chain reaction method by application of molecular biology, is proposed for future studies. Furthermore, a further study to measuring general infectivity including airborne fungi and viruses as well as airborne bacteria is necessary to assess potential severity of infection.

### Conclusion

The mean exposure level of airborne bacteria in pig buildings situated at South Korea was the highest in growing/fattening room followed by nursery room and gestation/farrowing room in terms of pig housing room (p<0.05). In seasonal aspect, the mean concentration of airborne bacteria measured in summer was the highest (p<0.05) and there was no significant difference among other seasons (p>0.05). The aerodynamic size of airborne bacteria in pig buildings was normally distributed and overall respirable airborne bacteria ranging between 0.6 and 4.7 μm (stage 3 to stage 6) were estimated to account for about 60% of the total airborne bacteria. The predominant species of airborne bacteria in pig buildings identified from this study were *Micrococcus* spp., *Brevibacillus* spp. and *G(+) Bacillus* spp. Based on the results obtained from this study, various measures, such as application of optimal ventilation rate and prompt manure treatment and wearing respirator for farmer’s health, to effectively to reduce concentration of airborne bacteria in pig buildings, should be undertaken especially in the growing/fattening room and summer season.

## Figures and Tables

**Figure 3:**
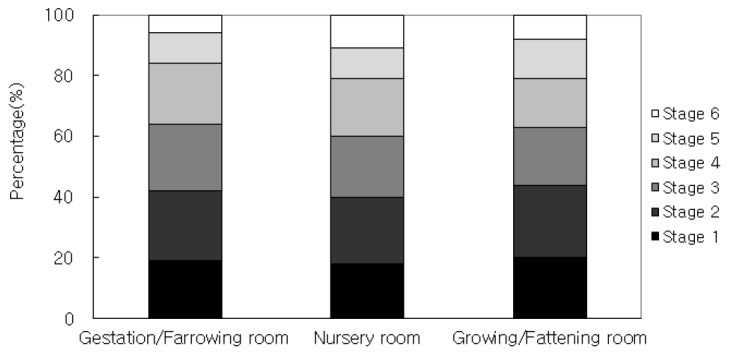
Size distribution of airborne bacteria in pig building according to type of pig housing room.

**Table 1:** Overview of pig buildings investigated in this study

No.	Location (province)	Area (m^2^)	No. of head (range)	Mean ventilation rate (m^3^/h)			

				Spring	Summer	Autumn	Winter
1	Gyeonggi	438	825–860	1.52	2.48	1.46	0.82
2		954	1,624–1,725	2.14	3.25	2.06	1.32
3		1,514	2,456–2,508	2.89	3.84	2.72	1.73
4	Gangwon	408	756–814	1.43	2.36	1.51	0.75
5		900	1,529–1,630	2.06	3.18	2.09	1.26
6		1,577	2,567–2,603	2.51	3.92	2.38	1.60
7	Chungbuk	488	924–951	1.84	2.64	1.72	1.08
8		831	1,427–1,489	2.22	2.93	2.06	1.41
9		1,508	2,463–2,482	2.68	3.85	2.51	1.84
1	Chungnam	344	641–683	1.26	2.37	1.37	0.61
2		983	1,706–1,742	1.63	2.98	1.52	1.21
3		1,419	2,302–2,357	2.14	3.74	2.01	1.53
4	Gyeongbuk	286	528–573	1.35	1.82	1.24	0.63
5		948	1,631–1,694	1.59	2.35	1.62	1.24
6		1,581	2,570–2,613	2.06	3.36	1.98	1.53
7	Gyeongnam	340	634–672	1.16	1.89	1.24	0.38
8		772	1,327–1,382	1.85	2.66	1.74	1.26
9		1,518	2,466–2,512	2.13	3.47	2.06	1.51
1	Jeonbuk	401	753–789	1.35	2.45	1.26	0.54
2		1,000	1,724–1,785	1.74	2.98	1.57	1.03
3		1,434	2,325–2,378	2.16	3.52	1.89	1.24
4	Jeonnam	348	658–682	1.24	2.12	1.36	0.78
5		877	1,527–1,551	1.67	3.13	1.51	1.21
6		1,487	2,425–2,451	2.34	4.02	2.18	1.51
7	Jeju	283	524–565	1.51	2.38	1.38	0.68
8		935	1,627–1,653	1.93	2.92	1.74	1.08
9		1,384	2,248–2,291	2.23	3.84	2.06	1.43

**Table 2:** Identification and detection rate of airborne bacteria in pig building according to type of pig housing room

Gestation/farrowing room	Nursery room	Growing/fattening room
*Micrococcus lylae* (23.8)	*Micrococcus lylae* (24.1)	*Micrococcus lylae* (20.2)
*Micrococcus luteus* (19.4)	*Micrococcus luteus* (15.3)	*Micrococcus luteus* (18.4)
*Brevibacillus choshinenesis* (7.2)	*Brevibacillus choshinenesis* (5.7)	*Brevibacillus choshinenesis* (8.2)
*Staphylococcus saprophytius* (4.8)	*Proteus mirabilis* (3.8)	*Aerococcus viridans* (6.4)
*Brevundimonas diminuta* (3.7)	Unidentified *G(+) Bacillus* (2.8)	Unidentified *G(+) Bacillus* (3.8)
Unidentified *G(+) Bacillus* (2.5)	Unidentified spp. (48.3)	Unidentified spp. (43.0)
Unidentified spp. (38.6)		

* ( ), detection rate, %.
